# Data integration in the era of omics: current and future challenges

**DOI:** 10.1186/1752-0509-8-S2-I1

**Published:** 2014-03-13

**Authors:** David Gomez-Cabrero, Imad Abugessaisa, Dieter Maier, Andrew Teschendorff, Matthias Merkenschlager, Andreas Gisel, Esteban Ballestar, Erik Bongcam-Rudloff, Ana Conesa, Jesper Tegnér

**Affiliations:** 1 Unit of Computational Medicine, Center for Molecular Medicine, Department of Medicine, Karolinska Institute and Karolinska University Hospital, Stockholm, Sweden; 2 Biomax Informatics AG, Munich, Germany; 3 Statistical Cancer Genomics, UCL Cancer Institute; Centre for Mathematics and Physics in the Life Sciences and Experimental Biology, University College London, London WC1E 6BT, UK; 4 Lymphocyte Development Group, MRC Clinical Sciences Centre, Imperial College London, London W12 0NN, UK; 5 Istituto di Tecnologie Biomediche (CNR), Unità Organizzativa di Bari, Via Amendola 122/D, 70126 Bari, Italy; 6 Chromatin and Disease Group, Cancer Epigenetics and Biology Program (PEBC), Bellvitge Biomedical Research Institute (IDIBELL), L'Hospitalet de Llobregat, Barcelona, Spain; 7 Department of Animal Breeding and Genetics, SLU Global Bioinformatics Centre, Swedish University of Agricultural Sciences, Uppsala, Sweden; 8 Computational Genomics Program, Centro de Investigaciones Príncipe Felipe, Valencia, Spain

## Abstract

To integrate heterogeneous and large omics data constitutes not only a conceptual challenge but a practical hurdle in the daily analysis of omics data. With the rise of novel omics technologies and through large-scale consortia projects, biological systems are being further investigated at an unprecedented scale generating heterogeneous and often large data sets. These data-sets encourage researchers to develop novel data integration methodologies. In this introduction we review the definition and characterize current efforts on data integration in the life sciences. We have used a web-survey to assess current research projects on data-integration to tap into the views, needs and challenges as currently perceived by parts of the research community.

## Introduction

Data integration is now a very commonly used notion in life sciences research. As of 2006 there were 1,062 papers explicitly mentioning "*data integration*" in their abstract or title, whereas this number has more than doubled in 2013 (2,365). However, there is still no unified definition of data integration, nor taxonomy for data-integration methodologies despite some recent efforts on this topic [1-5]. In February 2013, the FP7 STATegra project (http://stategra.eu/) and the COST Action SeqAhead (http://seqahead.eu/), two EU-funded initiatives on the bioinformatics of high-throughput data, organized in the city of Barcelona the "Workshop of Omics and Data Integration", with the aim of reviewing current technologies on omics data production and the available methods for their integrative analysis. The workshop consisted of contributed talks, sessions for open discussion and we included an on-line survey to investigate the current opinions of the research community on this topic. Three major conclusions were extracted from the Barcelona workshop. First, there is a clear need for revisiting the concepts of data integration and stating available resources in this field; second, it was advantageous to extend our survey to a broader audience of scientists in life sciences, and third the commitment of organizers to publish the discussed topics, contributions and outcome of the public survey in a relevant journal is an important driver to spearhead further discussion in the community. In this supplement we discuss these three conclusions in some detail. In this introductory article we review current definitions of data integration and describes it formally as the combination of two challenges: data discovery and data exploitation [5]. We briefly list major public efforts in creating resources (datasets, methods and workshops) for data integration. We also present the results of the extended community survey, which took place between February and March 2013 and on the basis of the survey we extract a couple of conclusions which warrant further elaboration in the community. Finally we introduce the contributions of the papers collected in this supplement within the context of the discussed data integration topics and stated community needs.

### Challenges of data integration in life sciences

**Figure 1:**
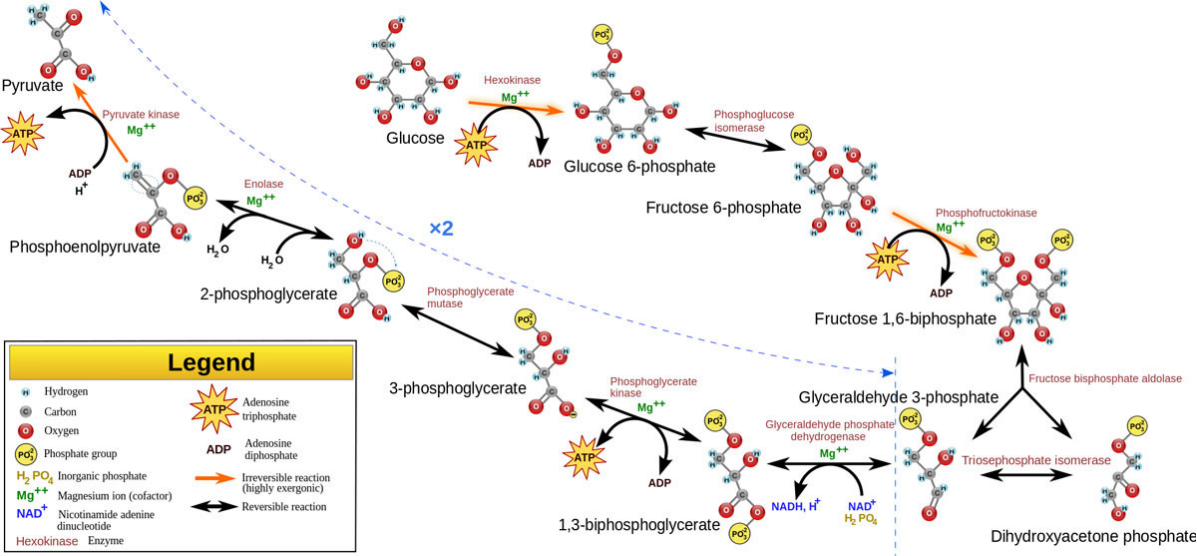
**Glycolysis**. Description of the ten reactions and their associated enzymes of the metabolic pathway (reproduced from http://en.wikipedia.org/wiki/File:Glycolysis2.svg).

Research in life sciences has the generic goal to identify the components that make up a living system (G1) and to understand the interactions among them that result in the (dys)functioning of the system (G2). Collection of biological data is therefore a method to catalogue the elements of life, but the understanding of a system requires the integration of these data under mathematical and relational models that can describe mechanistically the relationships between their components. We can illustrate the state of affairs on data integration in life science research using a simple example taken from metabolic modeling. Let us consider the glycolysis pathway (GLY), which consists of the conversion of glucose into pyruvate to release energy (see Figure 1 and [7]). In the study of GLY, G1 is considered "*to be known*" as there are a detailed set of genes, proteins and metabolites already described; however we are not yet certain that this list contains all involved elements, for example the list does not incorporate the epigenetic marks that may be associated to the regulation of GLY. When we consider G2, Figure 1 again depicts the current knowledge of the system and may erroneously imply that the system - defined as a set of interactions - is fully known. However, pathway elements and relations may be missing (see for instance the recent work on synthetic non-oxidative glycolysis [7]) and this representation does not allow us to determine completeness. Once more, the figure does not depict all the regulatory mechanisms involved or the rates of the reactions. This brings us to the first question of: *"What are the available data that can be used to fully characterize the GLY metabolic pathway*?"

The present situation is very fortunate since over the last decades several different types of data were generated and huge efforts were dedicated to create database repositories for different data-types where investigators were encouraged to deposit and share datasets associated with scientific publications. The benefits of this are twofold: on the one hand it enables or support researchers in reproducing and validating the analysis of other labs, and on the other hand it allows researchers to analyze data in novel ways and/or with different methodologies that were not originally considered by the team who generated the data. To illustrate this we investigated the availability of GLY-related datasets in Gene Expression Omnibus (GEO [8,9]) as an example of a major gene expression data repository and we readily made two observations. There exist a small number of datasets pertaining to the direct investigation of the GLY pathway, but the majority of microarray and NGS datasets contain information about the GLY pathway at the mRNA level. Moreover, it is possible to complement such information with enzyme kinetics data from databases such as BRENDA [10]. These observations bring us to the next questions. Once relevant data sources have been identified, "*How do we integrate all (or part of) the available datasets in order to improve our definition of the GLY system*?" and "*How do we re-use all this information when designing new and novel experiments*?"

All the above questions and challenges intuitively define the notion of "*data integration*".

### Data integration challenges

The term *data integration *refers to the situation where, for a given system, multiple sources (and possible types) of data are available and we want to study them integratively to improve knowledge discovery. In the GLY example system we could have two datasets describing the system, one containing information about gene expression at the mRNA level and the other describing the CpG DNA methylation profile. In several studies [11,12] where gene expression and DNA methylation data were available, the genome-wide relationships between DNA methylation and gene expression have been investigated in order to infer *generic *rules to questions such as: "Does DNA methylation regulation occurs at CpG islands and/or shores?", or "How does DNA methylation in promoters/gene-bodies/enhancers regulate gene expression?" [13]. These kinds of analyses have advanced our understanding of gene regulation by providing "generic rules yet with several exceptions" that associate epigenetic modifications with transcription [11,12]. For instance, as a general rule CpG methylation in promoters in mammals was found to be anti-correlated with gene expression, while CpG methylation in gene bodies in mammals was positively correlated; yet these generic rules are observed *as a trend*, but are not necessarily true for all genes and/or for all biological situations.

To understand the challenges of data integration it is first required to define the term. The term "*data integration*" first appeared from the need to access different databases with overlapping content to provide "*a redundancy free representation of information from a collection of data sources with overlapping content*" [14] which describes a need that appeared when the first databases were designed [15] and it was required to connect several of them: "*integration of multiple information systems aims at combining selected systems so that they form a unified new whole and give users the illusion of interacting with one single information system*". The aims of database integration were to make data more comprehensively available, and to increase the value of existing data by allowing previously difficult queries to be made upon it. Data mining (as a step in Knowledge Discovery in Databases [16,17]) is a major beneficiary from database integration. However this definition considers only *access *to data, and not *exploitation *of data, hence this definition of data integration is not fully applicable to life sciences research.

We define *data integration *as the use of multiple sources of information (or data) to provide a better understanding of a system/situation/association/etc; hence data integration, as defined here, is an action performed on a daily basis by most individuals, and a critical element in research.

Data integration in the life sciences becomes a more complex challenge considering the current "*data explosion*". This "*added*" challenge has been already been duly recognized; for instance in 2010 the National Research Council of the National Academies in US organized a workshop to "*explore alternative visions for achieving large-scale data integration in fields of importance to the federal government*" [5]. The workshop's aims and main results were reported in [5]; at the beginning of the document two main challenges associated with data integration were defined: *data discovery *and *data exploitation*. We followed the same structure in the present review and the next sub-sections briefly detail these challenges in the life sciences.

#### *Data discovery*

*Data source discovery *is defined as the identification of relevant data sources. Discovery of publicly available biological data sources is easy ("*just google it" *albeit with some exceptions, e.g. neuroscience [18]), whereas discovering the "*appropriate data*" is a more complicated task. One problem is the diversity of existing data types and formats, each one compliant to a different standard, which results in data heterogeneity and what has been called a "*loose federation of bio-nations*" [2]. The publication of specialized web databases has flourished in the last decade due to the relative ease of creation and maintenance and the reputation that it brings to the developers [2]. While specialized platforms may answer specific needs of the research community they may also introduce biases that affect data analysis. Two examples of this problem are the pathways and miRNA databases. The early 2000s witnessed the beginning of the generation of many pathway databases and their number has been increasing ever since, but has stabilized in the 2010s [2,4]. By 2013, *Pathguide *[19] reported a list of 547 biological pathways and molecular interaction related resources. These resources are not simply complementary, but often define similar signaling and metabolic pathways with different boundaries and components. This different specification is not irrelevant as many genome analysis methods are based on pathways and are therefore affected by how these are defined (see for instance [20] in this supplement). A second example relates to the storage of miRNA information [21]; this field has observed the development of generic purpose databases (e.g. miRBase and miRNAmap), many specialized databases (e.g. miRWalk, mirDB and Tarbase among others), and even standards for miRNA annotation [22]. In order to cope with this heterogeneity additional resources were developed such as catalogs of all available resources (e.g. *Pathguide *in pathways) and novel and larger databases developed in a joint effort between the developers of many older miRNA databases (see for instance RNAcentral [22]). We foresee two possible future scenarios: in the first one *developers of novel data-type resources, learning from previous experiences, will join efforts to consolidate data and create standards at earlier stages; in the second one we will accept redundant overlaps and solve them with data integration and knowledge management approaches*.

The rise of database resources certainly helps but does not solve entirely the problem of easy access to relevant data. An example is the Gene Expression Ombinus (GEO [8,9]); GEO (similarly to ArrayExpress [23]) is a data repository for microarray and NGS data that requires the data producers to submit data following the Minimum Information About a Microarray Experiment (MIAME) guidelines [24]. MIAME was originally designed to provide standards for microarray data sharing to ensure that "*data can be easily interpreted and that results from its analysis can be independently verified*" [24]; GEO requires that both raw and normalized data be available, samples are annotated (including experimental design) and laboratory and data processing protocols are described. Enforcing MIAME allowed many researchers to reexplore datasets from novel perspectives and "*more and more research is now built on the analysis of data that were not collected by the researchers themselves, and many of the extant data have not been utilized to their full potential*" [5]. However, annotation of experimental data in GEO still makes little use of controlled vocabularies (e.g ontologies), which is necessary for automated retrieval of relevant datasets for specific large-scale studies. Therefore finding datasets for a specific condition is possible, but using all samples associated to that condition without previous manual curation is still unfeasible.

We consider that the integration of Laboratory Information Management systems (LIMS) and/or Experiment Management Systems (see in this supplement [25]) in lab-life operations of omics data, and its standardization (such as the use of ontology-derived nomenclatures) and use in submission to public data repositories will smooth the path towards efficient data discovery and sharing.

#### *Data exploitation*

*Data exploitation *refers to the effective use of collective information to obtain new insights [5]. We can classify data exploitation according to the type of data used (similar or heterogeneous data types) or the information considered (all data points from all studies or summary results of individual studies, i.e. meta-analysis [26,27]). However, no classification will fully characterize contemporary research as researchers are blurring the boundaries by developing hybrid methodologies to optimize data analysis outcomes. We next develop some examples in current research.

If we consider datasets of similar data types, *meta-analysis *(that is, combining summary information from independent studies [26,27]) is a widely used statistical tool, as in many recent GWAS studies [28]. Importantly, we consider meta-analysis as a sub-type of data integration methodologies.

Data integration of heterogeneous data types is currently an active field of research where biostatisticians are constantly proposing hybrid approaches to improve data utilization and scientific discovery. Concepts such as the classification of data as "similar" or "heterogeneous" are still sometimes an open question [29] which clearly depends on the specific context. Hamid and collaborators define data as similar if they are from the "*same underlying source*" (e.g. all gene expression) and as heterogeneous if at least two fundamentally different data sources are involved (e.g. SNP and gene expression). Nevertheless other aspects such as technology may make integration complex, for example, when integrating RNA-seq and microarray based mRNA profiling. Following these definitions, and considering exploitation of datasets with heterogeneous data types involved (either across studies or within studies) then tools such as Co-Inertia Analysis [30,31], Generalized Singular Value Decomposition [32] and Integrative Bi-Clustering [33] among others are relevant. A comparison between these three methodologies in the integrative analysis of mRNA and protein abundance from a study of Plasmodium falciparum is included in this supplement [34]. In this supplement, Reverter et al. [35] propose a kernel PCA methodology that first selects the appropriate kernel for each data type and second combines the kernels from the different data types for a given statistical task.

Moreover, data exploitation in biological research involves not only actual datasets but also previous knowledge (sometimes referred to as Biological Domain Data [29]) which is captured in knowledge databases such as Gene Ontology [36] or the many biological pathway databases such as KEGG [37], or Reactome [38]. Gene Set Enrichment Analysis (GSEA, [39,40]) is a popular approach for integrating previous biological knowledge in the analysis of transcriptomics, which has been extended to other domains such as genomics and proteomics (e.g. [41] in this supplement) and the analysis of genomic regions (GREAT, [42]). Interestingly novel methods are still appearing that incorporate the biological domain knowledge also in the analysis of heterogeneous datasets. This supplement reviews the mathematical background of different methodologies that improve the integration of high-throughput transcriptomics and metabolomics data by incorporating prior knowledge in the form of gene sets and pathways [43].

### Brief overview of current approaches to data integration

Data integration is both a challenge and an opportunity and most certainly an increasing reality in genome research. Scientists have acknowledged that biological systems cannot be understood by the analysis of single-type datasets as the regulation of the system certainly occurs at many levels (see [29,44] and in this supplement [45]). Therefore projects have appeared aiming to investigate biological systems at several levels and *create large heterogeneous data-sets*. In several cases, such efforts ended in the design of *novel methodologies to analyze the data*. Furthermore *workshops and conference*s focused on the topic are starting to proliferate. These three aspects are detailed below.

### Data sources

The Human Genome Project [46,47] is probably the most well known biological project before 2000, but during the beginning of 21st century numerous other even more *data-intense *biological projects have been granted research funding. We aim to describe briefly a few of the most relevant projects, and prioritizing those where the resulting datasets are (or will be) publicly available. Other projects of interest not discussed here include the suite of Phantom Projects [48], TRANSFAC database [49] or the previously described GEO.

***1000 Genomes Project ***[50,51] aims to identify those generic genetic variants that have frequencies of at least 1% in the human population by sequencing many individuals with the novel NGS technologies. The project presented a technical challenge of how to store and manage not just the 1000 resulting genomes but the raw and processed data associated with them. The 1000 Genome Project is not as such a data integration driven project but certainly provides useful information on the identification of conserved regions and in GWAS studies.

***Encyclopedia of DNA Elements Project ***(ENCODE, [52-54]): considering that the genomes of several models organisms were nearly completed, ENCODE (*Homo Sapiens*), modENCODE (*C*. *elegans *and *D*. *melanogaster *[44]), and mouseENCODE (*Mus musculus *[55,56]) projects were launched with the common goal of identifying all functional elements within the genome, including "*protein-coding genes, non-protein-coding genes, transcriptional regulatory elements, and sequences that mediate chromosome structure and dynamics*" among them. These projects represent truly integration-based approaches which aim to characterize for a set of "animal models and/or tissues and/or cell lines" the profile of mRNA expression (e.g. RNA-seq, CAGE), histone marks and transcription factor binding profiling (ChIP-seq), DNA methylation (RRBS), chromatin conformation (e.g. ChIA-PET, 5C) and the location of active regulatory regions (DNAse-seq) among others. In September of 2012 the ENCODE consortium launched a synchronized publication effort with the preliminary analysis of the data.

***The Cancer Genome Atlas Project ***(TGCA): TGCA's major aim is to generate insights into the heterogeneity of different cancer subtypes by creating a map of molecular alterations for every type of cancer at multiple levels [57]. For instance the endometrial carcinoma has been characterized by mRNA, miRNA, protein, DNA methylation, copy number alterations and somatic chromosomal aberrations [58].

***Immunological Genome Project ***(ImmGen [59]) aims to characterize the mouse immunological system. ImmGen used microarrays to profile the mRNA of most immune cell types under carefully standardized conditions. Interestingly, ImmGen identified the project as a combined effort between immunologists and computational biologists, and is intended as a public resource. Not surprisingly, ImmGen has become a key resource in numerous investigations of the murine and human immune system research (*e*.*g*. [60]).

### Method development

Most of the previous data-intensive projects required the development of novel methodologies to analyze the data. Within ENCODE there has been a considerable effort to identify the relationship between combinations of histone marks and the activity state of DNA elements; Dynamic Bayesian Networks [61] have been used to classify intervals of the genome of K562 into specific classes (e.g. Protein Coding Transcription Start Sites) and more recently self-organizing maps [62] have been used for a similar purpose. Network analysis have also been addressed at ENCODE by the investigation of DNase-seq data, which allows the identification of active regulatory DNA elements, and its integration with Position Weight Matrixes to generate regulatory networks for each ENCODE cell-type [63,64]. To visualize networks circular plots were generated with Circos [65].

Immgen is the data-intensive project where the most advanced network inference methodology has been applied. In [66] authors developed Ontogenet to identify Transcription Factors (TF) acting as differentiation stage-specific regulators of mouse hematopoiesis. The methodology first identified 81 coarse- and 334 fine-grained expression modules, and secondly associates a set of TFs (among a pool of 580 candidates) to each one of these modules by defining the expression level of a module as the weighted linear combination of the associated regulatory TFs; the assignment uses a methodology similar to the Elastic Net [67] or Lasso, but adds penalty functions during the reconstruction of the network that prioritizes similarity (at the TF-module association stage) between cell lines that are closer in the lineage tree.

There is also a relevant need for the development of methodologies aiming to integrate omics and clinical data, both as network-based approaches [68,69] and as both network and data-driven approaches [70]. Overall, previous examples are just the tip of the iceberg of what has been developed, and we expect many more novel developments in the near future.

### Conferences, workshop and projects

Scientific meetings on data integration have proliferated in the last decade either as specialized stand-alone conferences or as part of a larger congress. To our knowledge the first International Workshop on Data Integration in the Life Sciences (DILS) took place in Germany in 2004, and last year, 2013, it was in Montreal; the Workshop aims "*to foster discussion, exchange, and innovation in research and development in the areas of data integration and data management for the life sciences*". This conference, which has a strong computational background [3], has consolidated as a major meeting point in data integration research. Also conferences such as the International Conference on Systems Biology has established workshops on integration-related topics such as metadata or data visualization (ICSB2013). The International Work-Conference on Bioinformatics and Biomedical Engineering of 2014 (IWBBIO2014) contains a special session devoted to *"integration of data, methods and tools in biosciences"*. Recent one-time events of interest are the session on Data Integration hosted at BioMedBridges in 2013; the Statistical Data Integration Challenges in Computational Biology: Regulatory Networks and Personalized Medicine Workshop organized at BIRS [71]; the Workshop in Genomic Data Integration 2013 [72] located at Imperial College, The Next NGS Challenge Conference: Data Analysis and Integration (Valencia, May 2013) and the High-throughput Omics and Data Integration Workshop in February 2013 from which this supplement originated.

### Canvassing the research community - a survey on data integration

From February to March 2013 we launched a web inquiry (see Additional file 1), that continued the survey initiated during the *Omics and Data Integration Workshop*, where we investigated major data-integration challenges for the research community in the field of life sciences. The results of this analysis are presented in this section.

#### *Survey: dissemination and biases*

By conducting a massive emailing effort among many institutions, the individuals that completed the survey (n = 125) more than doubled the number of registrations in relation to the workshop. Still, most participants were from Europe (80.8%) followed by US (5.6%), mostly from the academic sector (78.4%) and with major expertise in RNA-seq analysis (punctuation: 3/5) Complete DNA Sequencing (punctuation: 2.74/5). We obtained a proper balance between senior (37.6%) and junior (35.2%) researchers, and since the survey was answered by a limited number of individuals (125) we did not consider further stratification. Overall, we acknowledge that the present survey may not represent the views of the entire research community but it does highlight relevant questions and provides initial insights into the opinion of researchers dealing with data integration issues.

#### Survey: main results

An objective of the STATegra project (and also relevant to the wider scientific community) is to identify current and upcoming needs w.r.t data-analysis thus accelerating the development of novel integrative approaches. To investigate this, we included in the survey a question (4) to identify the major interests in single data types (see Additional file 1) for which individuals were able to select more than one answer. The following aspects of NGS data types caught the largest interest among the responders to the survey: RNA-seq (66.1%) and complete DNA-sequencing (36.3%). The second place was for clinical data (37.9%) followed by proteomics (35.5%). Most individuals were interested in the integrated analysis of multiple data types (72.8%) and this result was independent of participation in the Workshop in Omics Data Integration (Table 1) but significantly correlated with the researcher's expertise (p-value < 0.01).

**Table 1:** Scientific interest(s) of survey participants.

	ALL	Workshop participant	Not a participant
**Progress in experimental data production methods/technology**	25.60%	22.03%	28.79%
**Single data-type analysis methods**.	29.60%	37.29%	22.73%
**Multiple data-type integrated analysis**	72.80%	76.27%	69.70%
**Biomarker discovery**	35.20%	28.81%	40.91%
**Understanding of biological mechanisms**	56.80%	50.85%	62.12%
**Decision support for clinical care**	25.60%	16.95%	33.33%

This table summarizes Question 3 results (*Select the developments you are more interested in*). Survey participants were allowed to select more than one answer. The percentages of selected questions are shown for all participations and after stratification by their participation in the workshop

**Figure 2:**
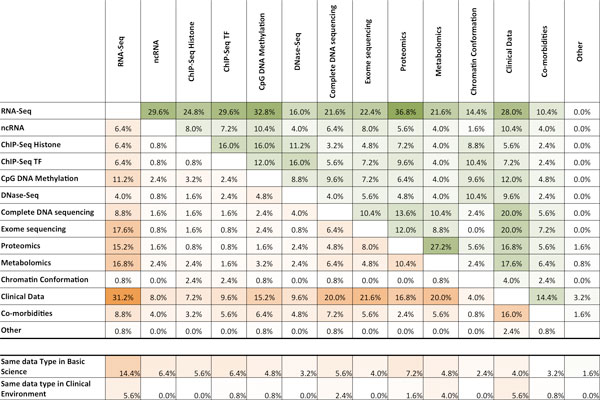
**Relevance of integration schemes**. (a) Each matrix location (*i*,*j*) shows the percentage of survey participants that selected as relevant the integration of data type *i *and data type *j *in basic (upper matrix) and clinical (lower matrix) research. (b) shows the percentage of participants that selected as relevant the integration of the same data type for the data types included in the list.

We next asked which integration schemes for two or more datasets were considered most relevant (Figure 2a upper matrix). We observed that the regulation of gene expression is a major goal and the integration of RNA-seq with all other data-types attracts great attention. Notably integration of clinical data was stated as very important, and this is relevant since this result does not particularly associate with the expertise and interests shown in Supp Table 1 in Additional file 2, but we believe reflects the continuous growth of translational research even in groups devoted to basic science. Integration of proteomics and RNA-seq was considered of high interest, together with a cluster formed by histone marks, transcription factor binding and CpG DNA methylation. We also investigated if these same integration priorities were maintained when thinking of clinical environments (Figure 2a, lower matrix). Clinical data and RNA-seq was the most frequently selected combination, also Clinical Data was highly associated to exome sequencing, complete DNA sequencing followed by metabolomics, proteomics and CpG DNA methylation. Not surprisingly co-morbidities was selected also as a very interesting data type for this setting.

Finally we observed that integration of same-type datasets was also highlighted (Figure 2b). Once more RNA-seq (14.4% basic science; 5.6% clinical environment) and clinical data (4.0%; 5.6%) were considered relevant (Figure 2c). Notably, only integration of several RNA-seq datasets is as highly prioritized as the integration of heterogeneous data types. Results were similar if the analysis was performed after stratification by individuals that "*participated or did not participate*" in the Workshop (results not shown).

##### Present tools in omics research

After stating the interest of the research community in data integration we surveyed their opinion in the availability of appropriate analysis tools. We designed a set of questions where "5" was associated to complete agreement, and "1" to complete disagreement. When considering the analysis of single data types there was an overall consensus (average score = 4.01) on the availability of proper tools, it was considered that most software was mainly available for researchers with a programming background (3.45). There was no clear consensus on the availability of user-friendly tools (2.72) but there was on the necessity of developing novel analysis tools in the field (4.55). The average opinion when asked about methods for integrative analysis of multiple data types was slightly different as there was no clear consensus on the availability of proper tools (2.57). Other aspects such as exclusivity of tools for programmers (3.84), existence of user-friendly software (2.21), and need of new analysis tools (4.72) had similar scores.

##### Future tools in omics research

When asked about what should be the major focus in the future the only and almost complete consensus was in the need of developing novel tools in explorative data analysis (4.45), causal discovery tools (4.50), knowledge-bases (4.29) and tools for making public data available and properly organized (4.51). A major requirement was the development of tools first as user-friendly software (4.60) and secondly as OpenSource software such as Bioconductor packages (4.16).

##### Funding and research

participants were asked where funding agencies would be required to invest in order to support the coming future of omics integrative analysis. Three questions with a 1 (least interesting) to 4 (most interesting) answer options were provided. Three funding goals were indicated as most relevant: *large publicly available data-sets *(2.35), *large data-sets from cohorts of selected diseases *(2.15) and new tools for data analysis (1.99). Still many participants indicated that other funding priorities were required such as education, a more focused tool development proposal on tools for integration with clinical data, data curation, and "*unification and standardization of all available omics data bases*".

##### Data standardization

Not included in the questionnaire design but mentioned by many responders in questions 16 (*Describe what you think is the most pressing/urgent/important research problem w.r.t data-integration*) and 17 (*Any comment you would like to add*?) was that data standardization is still an open issue. Standardization requirements were identified as two different but linked topics: the need to defined standard formats for every data type - which has been partially successfully managed by the several normalization efforts (e.g. MIAME), and the standardization of metadata. We acknowledge that, despite the enormous effort involved in providing annotated data repositories, the metadata included in many of them is still not sufficiently consistent or comprehensive enough to support large data approaches. *The editorial team agrees that resources must be committed to the developing and continuous support of public data repositories, while focusing not only in the challenges of storing the massive data files but also for more efficient annotation of the data involved. We believe that this goal will be facilitated by journal *policies requesting and controlling submission not only of data but also standardized metadata prior to publication.

### Open challenges and discussion

Data integration in the life sciences is not a new challenge, but it is a recurring one that has only recently been unfolded as a major challenge in part driven by technology development producing increasing amounts and type of data. However it is become increasingly clear that to be able to integrate across different types of is not only an opportunity but also a competitive advantage within the biological research community. While the availability of genomics data is reasonably well provided for by publicly accessible and well-maintained data repositories (with the relevant exception of clinical data), there is a need for improved (and novel) annotation standards and requirements in data repositories to enable better integration and reuse of publically available data.

The data exploitation aspect of data integration is probably the one that requires most attention, as it involves (1) the use of prior knowledge - and its efficient storage, (2) the development of statistical methods to analyze heterogeneous data sets and (3) the creation of data explorative tools that incorporate both useful summary statistics and new visualization tools.

We investigated in a survey with 125 responders what the most urgent questions of the research community are regarding data integration. Two relevant observations stand out: first, the need for user-friendly tools targeting integration of heterogeneous datasets; and secondly the relevance of translational medicine, as shown by the interest of incorporating clinical data in most integrative omic studies.

One aspect that we have not discussed in this editorial is that efficient data integration in life sciences may require the creation of novel research profiles. Most bioinformaticians engaged in the analysis of genomics data are either "*trained computer scientists or statisticians devoted to biology*", or "*trained biologists that were required to learn the basics of programming in order to dig deeper into their data*". While both are necessary and have pushed the field forward, it is increasingly recognized that the growth of computational biology requires the reformulation of the teaching system and the appearance of new wider syllabuses that cover all aspects of this interdisciplinary research filed in equivalent detail [73]). This is a major challenge, to raise a new generation of computer savvy researchers with a good understanding of the biology thus enabling development and application of relevant methods for intergration.

A second aspect we have not discussed is the impact of BIGdata analytics in the life sciences. The term BIGdata intuitively describes a situation present in many research fields: the amount of data generated by instruments is exploding, and in many cases doubling over short periods of time. Biology is not an exception: "*since *2008*, genomics data is outpacing Moore's Law by a factor of 4*" [74]. This situation results in the requirement for developing scalable infrastructures able to manage these quantities of data while making it available for efficient access and indexing. But more interestingly, big data have provided new ways to exploit data in many disciplines, such as economics (see *Data Economy*), business (as in Amazon or Google, [75]), high-energy physics [76] and even biology [77]. The main summary of BIGdata analysis is that even minor changes or low-level associations may be uncovered by the use of (very) large numbers of data points; therefore it remains to be seen how big data concepts will further reshape data integration in the life sciences.

A final aspect is that data integration is also seen as a commercial product and well-established companies (such as Ingenuity or Biomax) are competing with novel companies (such as Anaxomics or LifeMap) in a rapidly advancing field where the commercial edge is constantly being updated.

What is evident is that the era we are living in is nothing else than a paradise for integrative data analysis.

## Competing interests

No conflict of interest.

## Supplementary Material

Additional file 1 Survey details: *The needs & future in Omics & Data Integration*. Click here for file

Additional file 2 **Supplementary Table 1**. Interests (Question 4) and knowledge (Question 7) of participants on different research areas. Click here for file

## Declarations

This article has been published as part of *BMC Systems Biology *Volume 8 Supplement 2, 2014: Selected articles from the High-Throughput Omics and Data Integration Workshop. The full contents of the supplement are available online at http://www.biomedcentral.com/bmcsystbiol/supplements/8/S2.
