# Supplementary material for: Data integration in the era of omics: current and future challenges
Source: BMC Syst Biol. 2014 Mar 13;8(Suppl 2):I1. doi: 10.1186/1752-0509-8-S2-I1 (PMC4101704; doi:10.1186/1752-0509-8-S2-I1)
Supplement: Additional file 1 — Survey details: The needs & future in Omics & Data Integration. [file 1752-0509-8-S2-I1-S1.PDF]

Survey: The needs & future in Omics & Data Integration, 2013

Define yourself as part of the research community

*1. How would you define yourself?

- [ ] Senior
- [ ] Junior (up to PhD/less than 5 years experience)
- [ ] Basic science researcher
- [ ] Translational medicine researcher
- [ ] Clinical care
- [ ] IT
- [ ] Administration
- [ ] Software Developer
- [ ] Other, specify

*2. How would you define your employer?

- [ ] Academic
- [ ] Industrial: Pharma/Red Biotech
- [ ] Industrial: Chemical/White Biotech
- [ ] Industrial: Agrosciences/Green Biotech
- [ ] Industrial: Other
- [ ] Healthcare
- [ ] Editorial
- [ ] Other, specify

3. Select the developments you are more interested in (your aim by doing research):

- [ ] Progress in experimental data production methods/technology
- [ ] Single data-type analysis methods.
- [ ] Multiple data-type integrated analysis
- [ ] Biomarker discovery
- [ ] Understanding of biological mechanisms
- [ ] Decision support for clinical care
- [ ] Other, please specify

4. Select the data-type you are most interested in...

- [ ] RNA-Seq
- [ ] ncRNA
- [ ] ChIP-Seq Histone
- [ ] ChIP-Seq Transcription Factors
- [ ] CpG DNA-Methylation
- [ ] DNase-Seq
- [ ] Complete DNA sequencing
- [ ] Exome sequencing
- [ ] Proteomics
- [ ] Metabolomics
- [ ] Chromatin Conformation (ChIA-PET, HiC,...)
- [ ] Clinical Data
- [ ] Co-morbidities
- [ ] Other, Please specify

***5. Are you participating in the High-throughput Omics & Data Integration Workshop (http://www.seqahead.it/cost-bcn-2013/)**

- ( ) Yes
- ( ) No

6. Your main working place is located:

- ( ) Africa
- ( ) Asia
- ( ) Australia
- ( ) Europe
- ( ) North America
- ( ) South America
- ( ) If you want to specify continent & country

Where the tool developers and researchers should give the focus to...

7. How much do you know about...

	Nothing				Expert	N/A
RNA-Seq	◯	◯	◯	◯	◯	◯
ncRNA	◯	◯	◯	◯	◯	◯
ChIP-Seq Histone	◯	◯	◯	◯	◯	◯
ChIP-Seq Transcription Factors	◯	◯	◯	◯	◯	◯
CpG DNA Methylation	◯	◯	◯	◯	◯	◯
DNase-Seq	◯	◯	◯	◯	◯	◯
Complete DNA sequencing	◯	◯	◯	◯	◯	◯
Exome sequencing	◯	◯	◯	◯	◯	◯
Proteomics	◯	◯	◯	◯	◯	◯
Metabolomics	◯	◯	◯	◯	◯	◯
Chromatin Conformation (ChIA-PET, HiC,...)	◯	◯	◯	◯	◯	◯
Clinical Data	◯	◯	◯	◯	◯	◯
Co-morbidities	◯	◯	◯	◯	◯	◯

8. Which combination of data-types are more relevant in Basic Science? (multiple answers allowed)

	RNA-Seq	ncRNA	ChIP-Seq Histone	ChIP-Seq Transcription Factors	CpG DNA Methylation	DNase-Seq	Complete DNA sequencing	Exome sequencing	Proteomics	Metabolomics	Chromatin Conformation (ChIA-PET, HiC,...)	Clinical Data	Co-morbiditi
RNA-Seq	☐	☐	☐	☐	☐	☐	☐	☐	☐	☐	☐	☐	☐
ncRNA	☐	☐	☐	☐	☐	☐	☐	☐	☐	☐	☐	☐	☐
ChIP-Seq Histone	☐	☐	☐	☐	☐	☐	☐	☐	☐	☐	☐	☐	☐
ChIP-Seq Transcription Factors	☐	☐	☐	☐	☐	☐	☐	☐	☐	☐	☐	☐	☐
CpG DNA Methylation	☐	☐	☐	☐	☐	☐	☐	☐	☐	☐	☐	☐	☐
DNase-Seq	☐	☐	☐	☐	☐	☐	☐	☐	☐	☐	☐	☐	☐
Complete DNA sequencing	☐	☐	☐	☐	☐	☐	☐	☐	☐	☐	☐	☐	☐
Exome sequencing	☐	☐	☐	☐	☐	☐	☐	☐	☐	☐	☐	☐	☐
Proteomics	☐	☐	☐	☐	☐	☐	☐	☐	☐	☐	☐	☐	☐
Metabolomics	☐	☐	☐	☐	☐	☐	☐	☐	☐	☐	☐	☐	☐
Chromatin Conformation (ChIA-PET, HiC,...)	☐	☐	☐	☐	☐	☐	☐	☐	☐	☐	☐	☐	☐
Clinical Data	☐	☐	☐	☐	☐	☐	☐	☐	☐	☐	☐	☐	☐
Co-morbidities	☐	☐	☐	☐	☐	☐	☐	☐	☐	☐	☐	☐	☐
Other	☐	☐	☐	☐	☐	☐	☐	☐	☐	☐	☐	☐	☐

If "Other", please specify, or specify a combination not mentioned here

9. Which combination of data-types are more relevant in a Clinical Environment? (multiple answers allowed)

	RNA-Seq	ncRNA	ChIP-Seq Histone	ChIP-Seq Transcription Factors	CpG DNA Methylation	DNase-Seq	Complete DNA sequencing	Exome sequencing	Proteomics	Metabolomics	Chromatin Conformation (ChIA-PET, HiC,...)	Clinical Data	Co- morbiditi
RNA-Seq	☐	☐	☐	☐	☐	☐	☐	☐	☐	☐	☐	☐	☐
ncRNA	☐	☐	☐	☐	☐	☐	☐	☐	☐	☐	☐	☐	☐
ChIP-Seq Histone	☐	☐	☐	☐	☐	☐	☐	☐	☐	☐	☐	☐	☐
ChIP-Seq Transcription Factors	☐	☐	☐	☐	☐	☐	☐	☐	☐	☐	☐	☐	☐
CpG DNA Methylation	☐	☐	☐	☐	☐	☐	☐	☐	☐	☐	☐	☐	☐
DNase-Seq	☐	☐	☐	☐	☐	☐	☐	☐	☐	☐	☐	☐	☐
Complete DNA sequencing	☐	☐	☐	☐	☐	☐	☐	☐	☐	☐	☐	☐	☐
Exome sequencing	☐	☐	☐	☐	☐	☐	☐	☐	☐	☐	☐	☐	☐
Proteomics	☐	☐	☐	☐	☐	☐	☐	☐	☐	☐	☐	☐	☐
Metabolomics	☐	☐	☐	☐	☐	☐	☐	☐	☐	☐	☐	☐	☐
Chromatin Conformation (ChIA-PET, HiC,...)	☐	☐	☐	☐	☐	☐	☐	☐	☐	☐	☐	☐	☐
Clinical Data	☐	☐	☐	☐	☐	☐	☐	☐	☐	☐	☐	☐	☐
Co- morbidities	☐	☐	☐	☐	☐	☐	☐	☐	☐	☐	☐	☐	☐
Other	☐	☐	☐	☐	☐	☐	☐	☐	☐	☐	☐	☐	☐

If "Other", please specify, or specify a combination not mentioned here

Comments about available tools

10. How would you measure the tools available for single-data type analysis: 1 disagree to 5 agree (in relation to your data of interest).

	Disagree				Agree	N/A
There are good tools available						
Tools are only available for researchers with a programming background						
Tools are user friendly						
Development is required in the field						

11. How would you measure the tools available for integrative analysis:

	Disagree				Agree	N/A
There are good tools available						
Tools are only available for researchers with a programming background						
Tools are user friendly						
Development is required in the field						

12. Future tools should focus on:

	Disagree				Agree	N/A
Tools for Explorative Data Analysis						
Causal Discovery tools						
Making public data available and properly organized						
Knowledge-Bases						

13. Future tools should be delivered as:

	No				Yes	N/A
Bioconductor packages						
User-friendly Software (interface-based)						

14. Funding must be made available for the generation of:

[ ]	Large data-sets from cohorts of selected diseases	☐ N/A
[ ]	New and/or updated tools for data analysis	☐ N/A
[ ]	Other	☐ N/A
[ ]	Large publicly available data-sets (similar to ENCODE)	☐ N/A

15. If you want to provide specific definition to the option "Other" in the previous question

16. Describe what you think is the most pressing / urgent / important research problem w.r.t data-intergration.

17. Any comment you would like to add?

Final page.

18. This is the end of the survey. We would appreciate any further comments you would like to make.
Thanks for your time. Results of the questionary will be included in the BMC Systems Biology Supplement associated to the Workshop.
