# Supplementary material for: Data integration in the era of omics: current and future challenges
Source: BMC Syst Biol. 2014 Mar 13;8(Suppl 2):I1. doi: 10.1186/1752-0509-8-S2-I1 (PMC4101704; doi:10.1186/1752-0509-8-S2-I1)
Supplement: Additional file 2 — Supplementary Table 1. Interests (Question 4) and knowledge (Question 7) of participants on different research areas. [file 1752-0509-8-S2-I1-S2.docx]

**Supplementary Table 1. Interests (Question 4) and knowledge (Question 7) of participants on different research areas.**

|  | **Q4** | **Q7** |
| --- | --- | --- |
| **RNA-Seq** | 66.1% | 3.03 |
| **ncRNA** | 12.1% | 2.02 |
| **ChIP-Seq Histone** | 25.0% | 2.15 |
| **ChIP-Seq Transcription Factors** | 30.6% | 2.31 |
| **CpG DNA Methylation** | 22.6% | 2.22 |
| **DNase-Seq** | 17.7% | 2.14 |
| **Complete DNA sequencing** | 36.3% | 2.74 |
| **Exome sequencing** | 26.6% | 2.32 |
| **Proteomics** | 35.5% | 2.45 |
| **Metabolomics** | 28.2% | 2.31 |
| **Chromatin Conformation (ChIA-PET, HiC,…)** | 10.5% | 1.75 |
| **Clinical Data** | 37.9% | 2.51 |
| **Co-morbidities** | 12.1% | 1.72 |
